# High-fat diet accelerates pancreas pathology in the *Cel-HYB1* mouse model

**DOI:** 10.3389/fphys.2026.1924024

**Published:** 2026-09-25

**Authors:** Ranveig S. Brekke, Anny Gravdal, Renate V. Seierstad, Xunjun K. Xiao, Mark E. Lowe, Caroline S. Verbeke, Bente B. Johansson, Anders Molven, Karianne Fjeld

**Affiliations:** 1 Gade Laboratory for Pathology, Department of Clinical Medicine, University of Bergen, Bergen, Norway; 2 Department of Pediatrics, Washington University School of Medicine, St. Louis, MO, United States; 3 Department of Pathology, Oslo University Hospital Rikshospitalet, Oslo, Norway; 4 Department of Pathology, Institute of Clinical Medicine, University of Oslo, Oslo, Norway; 5 Mohn Center for Diabetes Precision Medicine, Department of Clinical Science, University of Bergen, Bergen, Norway; 6 Department of Pathology and Section for Cancer Genomics, Haukeland University Hospital, Bergen, Norway; 7 Department of Medical Genetics, Haukeland University Hospital, Bergen, Norway

**Keywords:** carboxyl ester lipase, chronic pancreatitis, disease risk, high-fat diet, mouse model

## Abstract

**Introduction:**

The *CEL* gene encodes the pancreatic enzyme carboxyl ester lipase. The human *CEL-HYB1* allele, a hybrid variant of *CEL* and its adjacent pseudogene *CELP*, is associated with a five-fold increased risk for chronic pancreatitis in European cohorts. Humanized *Cel-HYB1* mice develop chronic pancreatitis spontaneously albeit with a relatively mild presentation. The aim of this study was to further characterize the pancreatic phenotype in *Cel-HYB1* mice.

**Methods:**

We investigated *Cel-HYB1* animals by two complementary approaches. First, we analyzed compound heterozygous *Cel-16R/HYB1* mice where the second *Cel* allele carries the 16-repeat (*16R*) VNTR of the normal human *CEL* gene. Second, heterozygous and homozygous *Cel-HYB1* animals were fed either a high-fat diet or standard chow for 3 months. All animals were characterized with respect to growth, organ weights, pancreas pathology and glucose metabolism.

**Results:**

In the *Cel-16R/HYB1* model, body weight, pancreas weight and pancreatic histology were comparable to those of heterozygous *Cel-HYB1* mice. However, within each genotype female mice generally exhibited more pronounced pancreatic histopathological changes than males. Male *Cel-16R/HYB1* mice had larger fat depots at 6 months of age, and *Cel-16R/HYB1* animals of both sexes showed slightly improved glucose tolerance relative to heterozygous *Cel-HYB1* mice. When fed a high-fat diet, increased body weights were seen across all mouse groups without genotype-dependent differences. Under this condition, *Cel-HYB1* animals displayed more pronounced pancreatic histopathology than littermate controls. Notably, no consistent genotype-dependent differences in glucose tolerance were observed in *Cel-HYB1* mice under either high-fat diet or standard chow conditions.

**Conclusion:**

The humanized *Cel-16R* allele did not substantially modify the pancreatic phenotype of *Cel-HYB1* mice, although *Cel-16R/HYB1* animals exhibited somewhat improved glucose tolerance compared with heterozygous *Cel-HYB1* mice. In contrast, high-fat diet promoted exocrine pancreatic pathology, supporting a role for dietary factors as potential modifiers of disease severity in *CEL-HYB1*-associated chronic pancreatitis.

## 1 Introduction

Chronic pancreatitis (CP) is a complex disease which results in progressive destruction of the pancreas (Beyer et al., 2020), and both environmental factors and genetic variants are known to influence CP risk (Hegyi et al., 2020). The genetic risk factors can be divided into different mechanistic pathways driving pancreatitis onset and progression; namely the trypsin-dependent, misfolding-dependent and ductal pathways (Mayerle et al., 2019).

The misfolding dependent pathway drives disease development by impaired enzyme secretion, intracellular protein retention and increased endoplasmic reticulum (ER) stress (Sahin-Toth, 2017; Mayerle et al., 2019). CP risk alleles involved in this pathway encode enzymes produced by the acinar cells of the pancreas, including cationic trypsinogen (PRSS1), carboxypeptidase A1 (CPA1), pancreatic lipase (PNLIP) and carboxyl ester lipase (CEL) (Kereszturi et al., 2009; Schnur et al., 2014; Hegyi and Sahin-Toth, 2019; Fjeld et al., 2022; Zhu et al., 2023; Gravdal et al., 2025).

CEL is a digestive enzyme stimulated by bile salts, and it hydrolyzes cholesterol esters, dietary fat and fat-soluble vitamins (Lombardo et al., 1978; Lombardo and Guy, 1980; Hui and Howles, 2002). The *CEL* gene contains 11 exons where the last exon includes a variable number tandem repeat (VNTR) region (Johansson et al., 2018). The number of VNTR segments in humans varies greatly, but 16 repeats is by far the most common number (Torsvik et al., 2010; Fjeld et al., 2016; Johansson et al., 2018).

We have previously reported that a copy number variant of *CEL*, named *CEL-HYB1*, confers increased risk for developing CP (Fjeld et al., 2015). In German and French cohorts of nonalcoholic CP patients, *CEL-HYB1* was five-fold more frequent than in healthy controls. This association was replicated in Hungarian adult CP cases but not in Polish pediatric cases (Oracz et al., 2019; Berke et al., 2024). The effect of *CEL-HYB1* may therefore vary across European populations, possibly due to differences in underlying haplotypes (Cassidy et al., 2020; Berke et al., 2024). Interestingly, screening of three Asian cohorts with idiopathic CP did not identify the *CEL-HYB1* allele in any subject, suggesting that it represents an ethnicity-specific risk factor for the disease (Molven et al., 2016; Zou et al., 2016).

*CEL-HYB1* is a hybrid allele formed between *CEL* and its neighboring pseudogene *CELP*, comprising exons 1–10 from *CEL* and exon 11 from *CELP.* As a result, *CEL-HYB1* contains only three VNTR repeats (Fjeld et al., 2015), and cellular studies have demonstrated that *CEL-HYB1* follows the misfolding-dependent pathogenic pathway of genetic risk in chronic pancreatitis (Fjeld et al., 2015; Cassidy et al., 2020; Dalva et al., 2020; Tjora et al., 2021). Furthermore, two *Cel-HYB1* mouse models have been described by both our group and others, using different methodological approaches (Fjeld et al., 2022; Mao et al., 2022). These models exhibited a similar phenotype of spontaneous but slowly developing CP, confirming the pathogenic role of human *CEL-HYB1* and implicating that *Cel-HYB1* mice could be suitable for investigating gene–gene and gene–environment interactions in CP.

In the present study, we intended to extend our understanding of how *CEL-HYB1* predisposes to pancreatic disease. To this end, we investigated our *Cel-HYB1* mouse model by two different approaches: *(i)* we created a compound heterozygous *Cel-16R/HYB1* mouse to directly mimic the genetic constellation of human *CEL-HYB1* carriers and *(ii)* we stressed the *Cel-HYB1* animals by exposing them to a high-fat diet (HFD).

## 2 Materials and methods

### 2.1 Generation of mouse strains

The *Cel-HYB1* and *Cel-16R* mouse strains were constructed by genOway (Lyon, France). The strains were established and kept on a C57BL/6N genetic background (Charles River Laboratories, Lyon, France). Details are provided in (Fjeld et al., 2022; Brekke et al., 2024). In brief, the mouse *Cel* VNTR was replaced by the human *CEL-HYB1* VNTR and the normal *CEL* VNTR (harboring 16 repeats) in the *Cel-HYB1* and *Cel-16R* models, respectively. To create *Cel-16R/HYB1* mice, homozygous *Cel-HYB1* males were bred with heterozygous *Cel-16R* females. For sequences and nomenclature of the mouse/human *Cel/CEL* genes, see (Fjeld et al., 2022).

### 2.2 Ethics and animal maintenance

The mice were housed and cared for at the Laboratory Animal Facility, Faculty of Medicine, University of Bergen, Norway, which is accredited by the Association for Assessment and Accreditation of Laboratory Animal Care International. Breeding and experiments were approved by the Norwegian Animal Research Authority with the approval ID FOTS28946. The experiments were conducted according to the European Convention for the Protection of Vertebrates Used for Scientific Purposes.

All mice were housed on a 12-h light/dark cycle with *ad libitum* access to water and food, and both male and female animals were studied. The number of animals, sex, age and genotype used in each experiment are shown in the figures. At experimental endpoints, mice were euthanized via gradual CO_2_ inhalation (30–70% chamber volume/min, starting with room air) for at least 3 min, in compliance with institutional guidelines. Blood samples were immediately collected via cardiac puncture. Tissue samples were harvested as indicated and weighed before fixed in 4% formalin. For the HFD study, the control group was fed a standard chow diet (Ssniff, V1534-703), while the HFD group were fed a diet containing 60% fat (Brogaarden, D12492).

### 2.3 Genotyping

DNA was extracted from ear punches using E.Z.N.A Tissue DNA Kit (Omega Bio-Tek, D3396-02). Genotyping was performed by using a Multiplex PCR Kit (Qiagen, 206143) and involved three PCR assays: one general reaction that differentiated between wildtype, heterozygous and homozygous knock-in mice, and a specific PCR assay for either the *Cel-HYB1* or *Cel-16R* knock-in allele (**Supplementary Figure 1**).

Primers used for the general PCR assay were 5`-GCA AAC TTC TTA TTT ATC CTC AAG CCT TGG-3` (forward) and 5`-GTT ATC GTC TTA GTG ATG TCC AGG TAG TTG C-3` (reverse). Amplicon sizes for the wildtype and knock-in alleles were 303 and 394 bp, respectively. Primers used for the *Cel-HYB1*- specific assay were 5`-CGC AGA GCT GTC CAG GAG CAC G-3’ (forward) and 5`-CTG TGG AGG GCA TGG AAC T-3’ (reverse). The amplicon size was 278 bp. Primers used for the *Cel-16R*-spesific assay were 5`-CCA CCA TGA GTC CAA TGA TTG CAC C-3` (forward) and 5`-GGT GGC CTC CTG GTC GGT CAC T-3` (reverse). The amplicon size was 286 bp.

### 2.4 Histology and evaluation of pathology

Formalin-fixed (4%) and paraffin-embedded (FFPE) pancreatic tissue samples were sectioned (4-5 µm) and stained with hematoxylin and eosin (HE) using a standard protocol. For both the *Cel-16R/HYB1* and HFD study, histological changes were evaluated on HE sections and scored blindly (i.e. without access to genotype, age or sex) by C.S.V., R.V.S. and K.F. Inflammation, fatty replacement and acinar atrophy were assessed, and for each parameter, a score of normal (0), mild (1) or moderate (2) changes was given (**Table 1** and **Table 2**). Fatty replacement was defined as the presence of both intralobular and extralobular adipocytes.

**Table 1:** Histology scores of pancreatic sections from *Cel-HYB1* and *Cel-16R/HYB1* mice.

Males				Females			
Scores	Genotypes			Scores	Genotypes		
	*Cel ^+/+^*	*Cel ^+/HYB1^*	*Cel ^16R/HYB^*		*Cel ^+/+^*	*Cel ^+/HYB1^*	*Cel ^16R/HYB^*
	n=2	n=10	n=6		n=2	n=9	n=7
Inflammation				Inflammation			
0	2	8	5	0	2	5	4
1		2	1	1		3	3
2				2		1	
Fatty replacement				Fatty replacement			
0	2	6	3	0	2	1	3
1		3	3	1		7	4
2		1		2		1	
Acinar atrophy				Acinar atrophy			
0	2	5	5	0	2	4	3
1		5	1	1		5	4
2				2			

**Table 2:** Histology scores of pancreatic sections from *Cel-HYB1* mice on chow and high-fat diet.

Chow: males				Chow: females			
Scores	Genotypes			Scores	Genotypes		
	*Cel^+/+^*	*Cel^+/HYB1^*	*Cel^HYB1/HYB1^*		*Cel^+/+^*	*Cel^+/HYB1^*	*Cel^HYB1/HYB1^*
	n = 2	n = 6	n = 6		n = 2	n = 6	n = 5
Inflammation				Inflammation			
0	2	6	5	0	2	6	5
1			1	1			
2				2			
Fatty replacement				Fatty replacement			
0	2	5	6	0	2	6	5
1		1		1			
2				2			
Acinar atrophy				Acinar atrophy			
0	2	6	6	0	2	6	5
1				1			
2				2			
HFD: males				HFD: females			
Scores	Genotypes			Scores	Genotypes		
	*Cel^+/+^*	*Cel^+/HYB1^*	*Cel^HYB1/HYB1^*		*Cel^+/+^*	*Cel^+/HYB1^*	*Cel^HYB1/HYB1^*
	n = 6	n = 6	n = 6		n = 6	n = 6	n = 6
Inflammation				Inflammation			
0	5	5	4	0	5	6	5
1		1	1	1	1		
2	1		1	2			1
Fatty replacement				Fatty replacement			
0	4	3	2	0	6	4	2
1	2	3	4	1		2	4
2				2			
Acinar atrophy				Acinar atrophy			
0	6	6	5	0	6	6	5
1			1	1			1
2				2			

### 2.5 Immunohistochemistry

Immunohistochemistry was carried out as previously described (El Jellas et al., 2018). Before staining with the primary antibodies anti-CEL (Sigma, HPA052701; 1:200), the tissue slides were boiled at 120°C for 1 min in a Tris-EDTA buffer, pH 9.0 (Thermo Fischer Scientific, 00-4956-58) for antigen retrieval before treatment with serum-free protein block (Agilent, X0909). For primary antibody detection, an anti-rabbit probe (BioCare Medical, RP531H) was used followed by an HRP-conjugated polymer (Biocare Medical, RH531H) and visualization with 3,3`-diaminobenzidine (Agilent). The sections were counterstained with hematoxylin (Agilent, S3301) and scanned using Nano Zoomer XR (Hamamatsu Photonics). Images were obtained by Aperio ImageScope (Leica Biosystems).

### 2.6 Blood glucose measurements

Random glucose measurements were performed in the morning (09.00-12.00) with the animals having *ad libitum* access to water and food. Blood was collected by a small incision in the tail and blood glucose was measured using a FreeStyle Lite glucometer (Abbott). Intraperitoneal glucose tolerance tests (IPGTT) were carried out as previously described (Fjeld et al., 2022). Area under the curve (AUC) calculation was carried out using GraphPad Prism 11.0.0.

### 2.7 Serum amylase activity

Blood was collected through post-mortem cardiac puncture and transferred to tubes coated with a clot activator (Sarstedt, 16.440.100). After 20 min incubation in room temperature, the blood samples were centrifuged for 10 min (3000 x g, 4°C) and the serum pipetted into new tubes and stored at -80˚C until further use. Amylase activity measurements were carried out as previously described (Fjeld et al., 2022) using the substrate 2-chloro-*P*-nitrophenyl-a-D-maltotrioside (Pointe Scientific, Medtest Dx, A7564-120). Amylase activity (rate of substrate cleavage) was expressed as mOD/min.

### 2.11 Statistics

Results are presented as mean ± standard deviation (SD) or standard error of the mean (SEM), as indicated. Differences between two groups were analyzed using Fisher exact or a two-tailed unpaired t-test, while comparisons among three groups were performed using one-way ANOVA followed by appropriate *post hoc* tests.

## 3 Results

### 3.1 The *Cel-16R/HYB1* mouse study

The normal mouse *Cel* gene contains only three VNTR repeats (Fjeld et al., 2022), whereas the most common human *CEL* VNTR has 16 repeats (Johansson et al., 2018). Previous studies have shown that the *Cel-16R* mouse has a pancreatic phenotype similar to controls, confirming that a VNTR extension in mice does not by itself result in pancreatic disease (Brekke et al., 2024; Gravdal et al., 2025). Here we studied compound heterozygous *Cel-16R/HYB1* (*Cel^16R/HYB1^*) mice to see if the combined presence of the *CEL-16R* and *CEL-HYB1* alleles would influence the pancreatic phenotype.

#### 3.1.1 Development and growth

*Cel^16R/HYB1^* animals were monitored for up to 12 months and compared with heterozygous *Cel-HYB1* (*Cel^+/HYB1^*) in-cage littermates. The body weights of *Cel^+/HYB1^* and *Cel^16R/HYB1^* mice were measured bi-weekly. *Cel^16R/HYB1^* male mice tended to have higher body weights and *Cel^16R/HYB1^* females lower body weights than *Cel^+/HYB1^* animals. However, these differences did not reach statistical significance (**Figure 1A**). Pancreatic, white adipose, and brown adipose tissue masses were assessed relative to body weight at 6 and 12 months. No statistically significant differences were observed (**Figures 1B, C**). The only exception was epididymal and subcutaneous fat masses, which were significantly increased in *Cel^16R/HYB1^* male mice at 6 months (**Figure 1C**). Consistent with the relative pancreas weight data, male *Cel^16R/HYB1^* mice showed a tendency toward higher absolute pancreas weights at 12 months; however no statistically significant differences in absolute pancreas weights were observed between genotypes for either sex at 6 or 12 months of age (**Supplementary Figure 2**).

**Figure 1:**
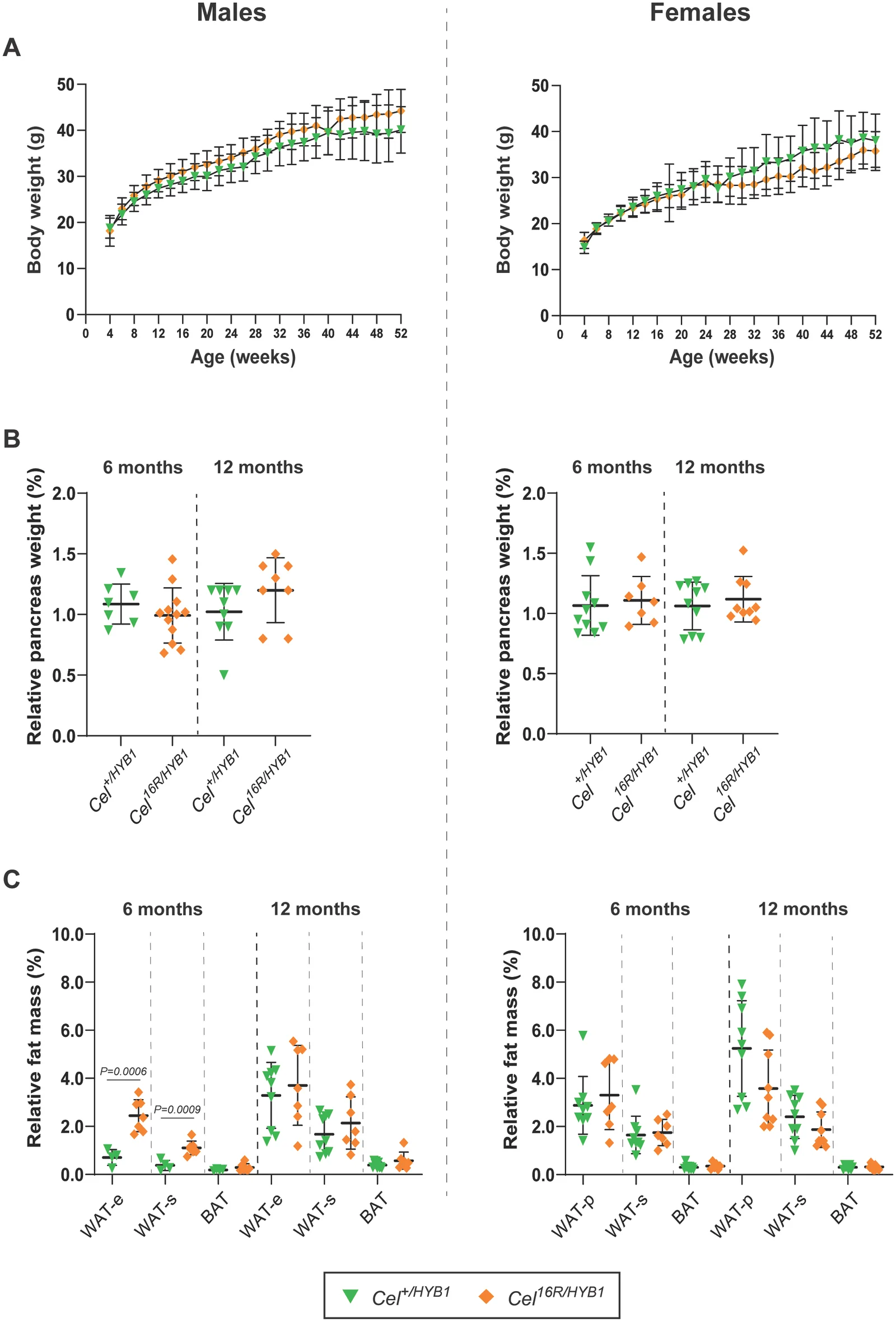
Body weight, pancreas weight and fat mass in *Cel^+/HYB1^* and *Cel^16R/HYB1^* male and female mice. **(A)** Body weight development in mice from 4 to 52 weeks of age. **(B)** Relative pancreas weight and **(C)** relative fat mass in mice at 6 and 12 months of age, expressed as percentage of body weight. Individual values with mean (horizontal bar) ± SD are shown. WAT, white adipose tissue; e, epididymal; p, parametrial; s, subcutaneous; BAT, brown adipose tissue. *P*-values are listed for statistically significant differences between groups.

#### 3.1.2 Chronic pancreatitis development

Pancreatic histology of *Cel^+/HYB1^* and *Cel^16R/HYB1^* mice was evaluated by histological scoring of HE-stained pancreatic tissue sections at 12 months of age (**Table 1**). Both genotypes had mild histopathological changes with inflammation, fatty replacement, and acinar atrophy observed in subsets of animals from both groups. Interestingly, female mice generally exhibited more pronounced histopathology than male mice, irrespective of genotype. In addition, fatty replacement was observed in more female *Cel^+/HYB1^* mice than *Cel^16R/HYB1^* females, whereas atrophy was more often seen in male *Cel^+/HYB1^* mice than in *Cel^16R/HYB1^* males. Inflammation appeared similar between the two genotypes. Representative pancreatic sections from male and female mice are shown in **Figure 2A**. Our data support the conclusion that wild-type CEL VNTR length has limited impact on the pancreatic phenotype of *Cel-HYB1* mice under basal conditions.

**Figure 2:**
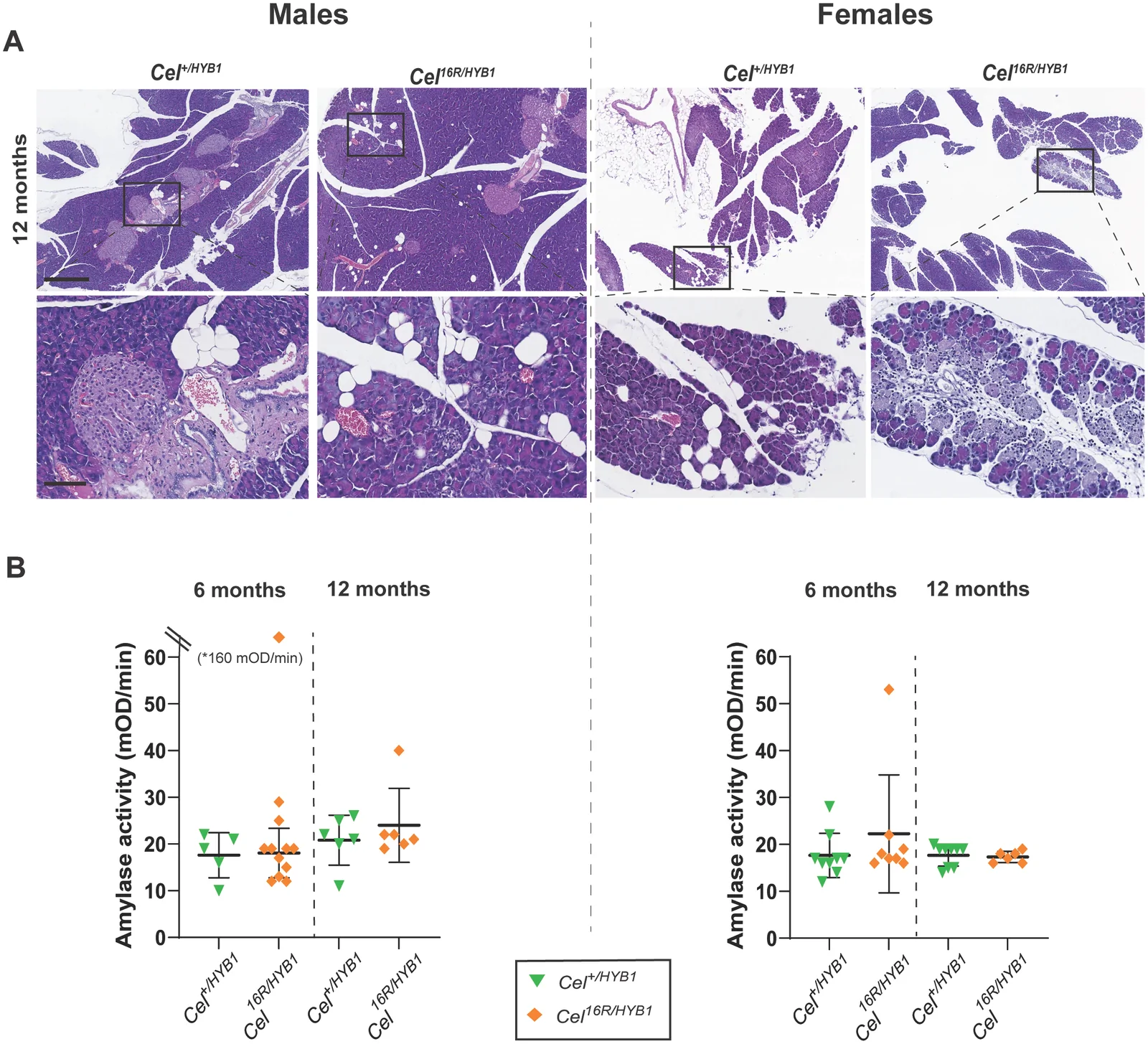
Pancreas histology and amylase activity in *Cel^+/HYB1^* and *Cel^16R/HYB1^* male and female mice. **(A)** Representative HE-stained pancreatic sections from mice at 12 months of age. Scale bars are 500 µm (for images with low magnification) and 100 µm (high magnification). **(B)** Serum amylase activity measured in blood samples from mice at 6 and 12 months of age. Individual values with mean (horizontal bar) ± SD are shown.

No statistical difference was observed in amylase activity between the genotypes. However, a few *Cel^16R/HYB1^* mice stood out with a large increase in amylase activity levels at 6 and 12 months (**Figure 2B**).

#### 3.1.3 Pancreatic endocrine function

We monitored endocrine function of the *Cel^+/HYB1^* and *Cel^16R/HYB1^* mice by measuring random blood glucose bi-weekly until 12 months. There was no indication of diabetes development at any timepoint (data not shown). We also performed IPGTT at 6 and 12 months (**Figure 3A**). There was a general trend that *Cel^16R/HYB1^* animals were slightly more glucose-tolerant than *Cel^+/HYB1^*, and this difference reached statistical significance for both male (6 months) and female mice (6 and 12 months) when assessed as AUC (**Figure 3B**).

**Figure 3:**
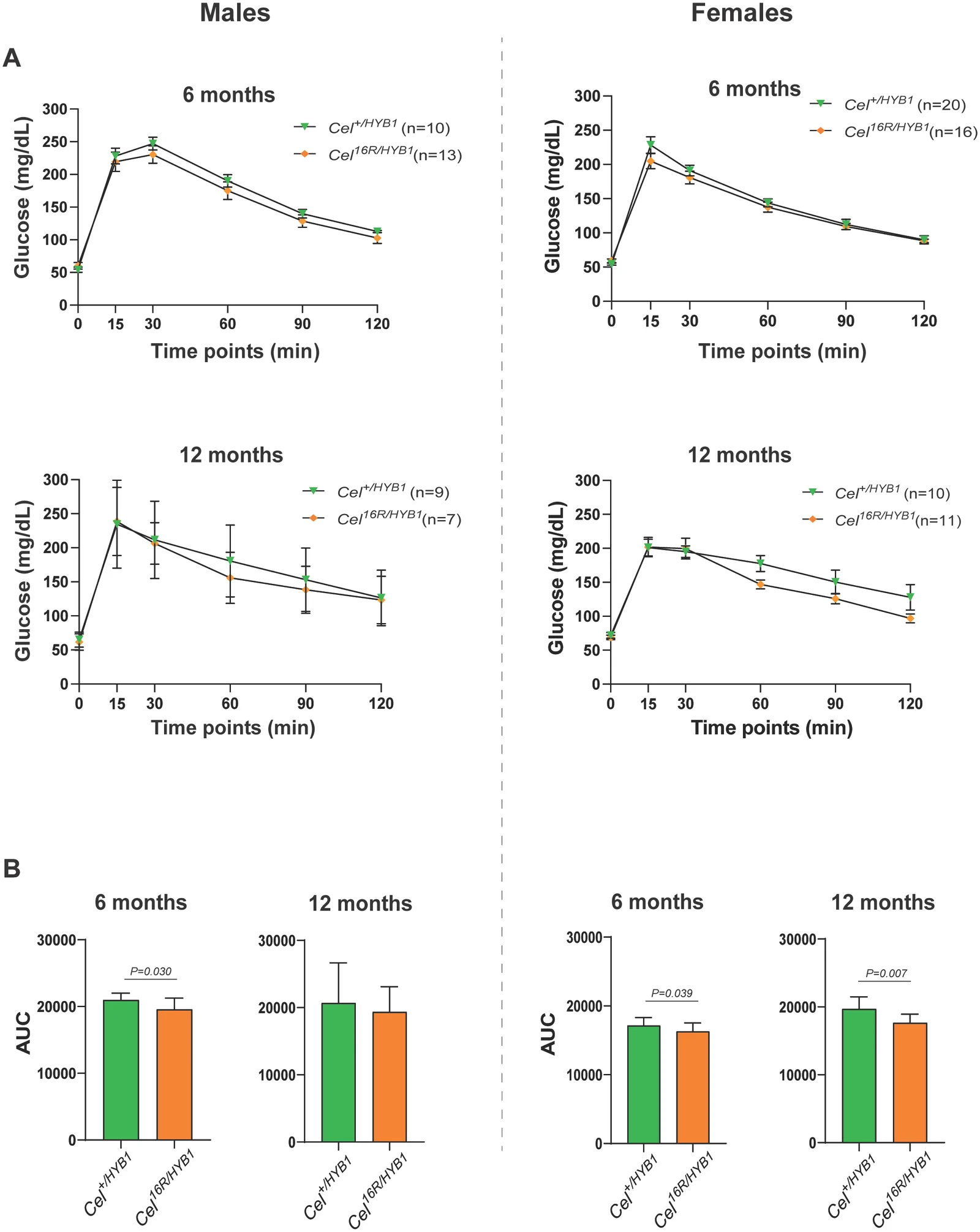
Glucose homeostasis in *Cel^+/HYB1^* and *Cel^16R/HYB1^* male and female mice. **(A)** Intraperitoneal glucose tolerance test of mice at 6 and 12 months. **(B)** Area under the curve (AUC) for the corresponding graphs presented in **(A)**. Results are shown as mean ± SEM. *P*-values are listed for statistically significant differences between groups.

### 3.2 The *Cel-HYB1* high-fat diet study

Apart from highly penetrant variants such as *PRSS1*, most genetic risk variants for CP require additional environmental or genetic factors to trigger disease development (Hu et al., 2017; Kleeff et al., 2017). We have previously suggested that cigarette smoking and pancreas divisum may accelerate disease progression in *CEL-HYB1*-associated CP (Tjora et al., 2021). Here, we exposed *Cel-HYB1* mice to high-fat diet (HFD) to assess whether metabolic stress and increased body weight influences the exocrine or endocrine phenotype in these animals. Control (*Cel^+/+^*), heterozygous (*Cel^+/HYB1^*) and homozygous (*Cel^HYB1/HYB1^*) male and female mice were fed standard chow in addition to maternal milk until three weeks of age. Then, the animals were separated into two groups, where one group continued on chow whereas the other group was given HFD (**Figure 4A**). We started monitoring body weight when the mice were six weeks old and continued biweekly until the experimental endpoint (16 weeks). At 15 weeks, IPGTT was performed.

**Figure 4:**
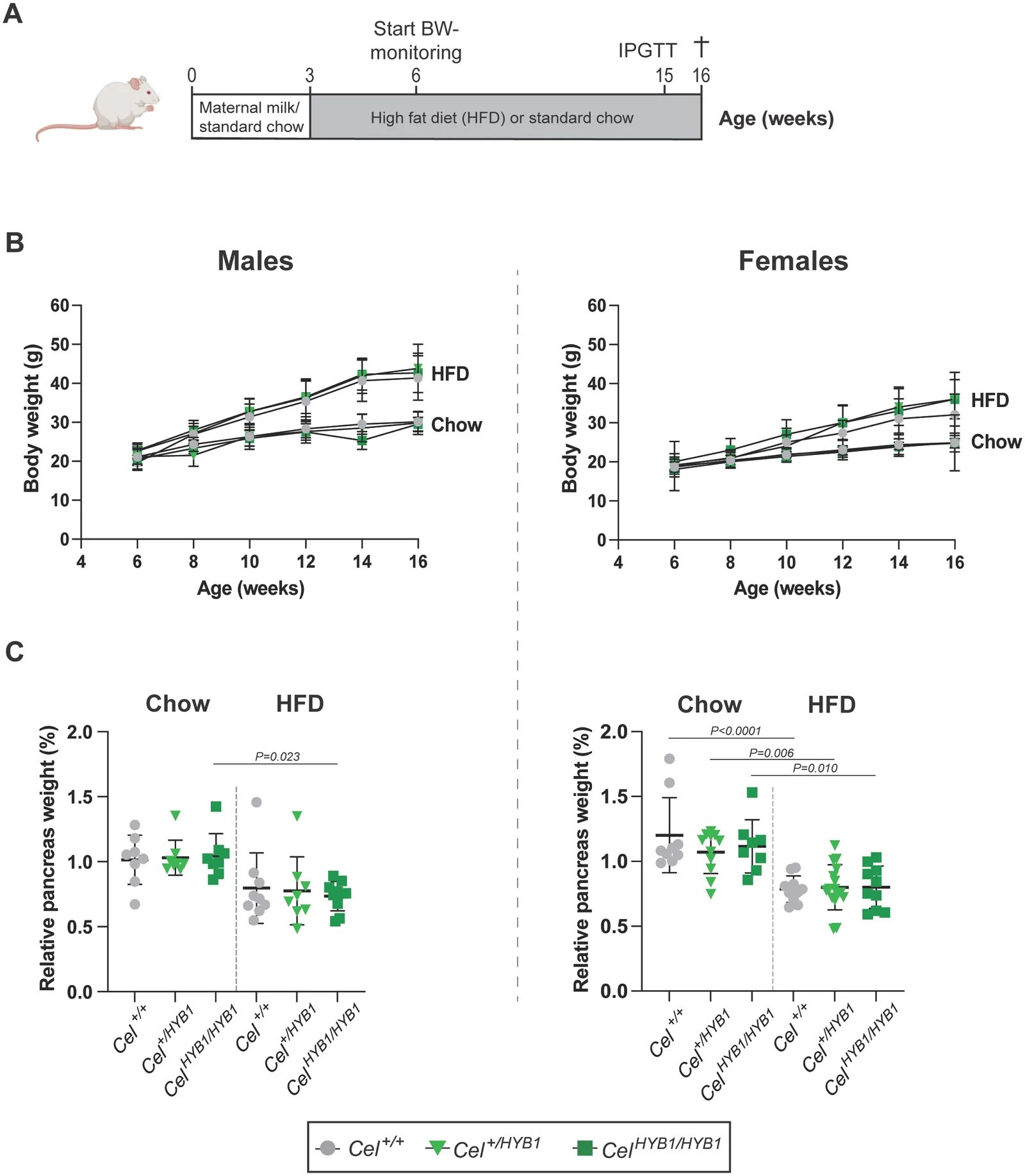
Body weight development in *Cel-HYB1* mice on HFD. **(A)** Overview of the experiment. Mice were fed maternal milk supplemented with a control diet (chow) until 3 weeks of age. After weaning, the mice were maintained on chow or switched to HFD for 12 weeks. Body weight (BW) monitoring started at 6 weeks of age. At 15 weeks, the mice underwent an IPGTT before being sacrificed at 16 weeks. **(B)** Body weight development in *Cel^+/+^*, *Cel^+/HYB1^* and *Cel^HYB1/HYB1^* male and female mice on chow diet or HFD. **(C)** Relative pancreas weight (percentage of body weight) in *Cel^+/+^*, *Cel^+/HYB1^* and *Cel^HYB1/HYB1^* male and female mice on chow diet or HFD. Individual values are shown with mean (horizontal bar) ± SD. *P*-values are listed for statistically significant differences between groups.

#### 3.2.1 Body and organ weight

As expected, HFD-fed *Cel^+/+^*, *Cel^+/HYB1^* and *Cel^HYB1/HYB1^* mice showed increased body weight when compared with chow-fed mice (**Figure 4B**), However, no difference was observed between genotypes. Pancreas mass was assessed at 16 weeks, and chow-fed mice had higher relative weights than the HFD-fed mouse groups (**Figure 4C**). However, genotype did not influence pancreas mass as the relative weights within each diet group and for each sex were similar for *Cel^+/+^*, *Cel^+/HYB1^* and *Cel^HYB1/HYB1^* animals.

#### 3.2.2 The effect of HFD on the exocrine pancreas

Histopathological assessment of the HE-stained pancreatic tissue sections obtained from the different experimental groups was performed. Most *Cel-HYB1* mice on chow showed normal pancreas morphology, except for two male animals with signs of inflammation and fatty replacement (**Table 2** – upper section). In contrast, the majority of male and female *Cel-HYB1* mice on HFD exhibited histological changes including inflammation, fatty replacement and acinar atrophy (**Table 2** – lower section). The alterations were more pronounced in homozygous *Cel-HYB1* mice than in heterozygous littermates. Notably, three of 12 HFD-fed *Cel^+/+^* mice presented with similar histopathological features, indicating that HFD alone may induce pancreatic tissue changes in control mice. Representative pancreatic sections from HFD-fed male mice are shown in **Figure 5A**. Overall, there was a higher proportion of affected mice in the *Cel-HYB1* HFD group compared to controls (15/24 vs 3/12; OR = 5.0, *P* = 0.075), although the difference did not reach statistical significance due to limited number of animals. Serum amylase activity was not significantly elevated by HFD or standard chow diet, neither in wildtype nor in *Cel-HYB1* mice (**Figure 5B**).

**Figure 5:**
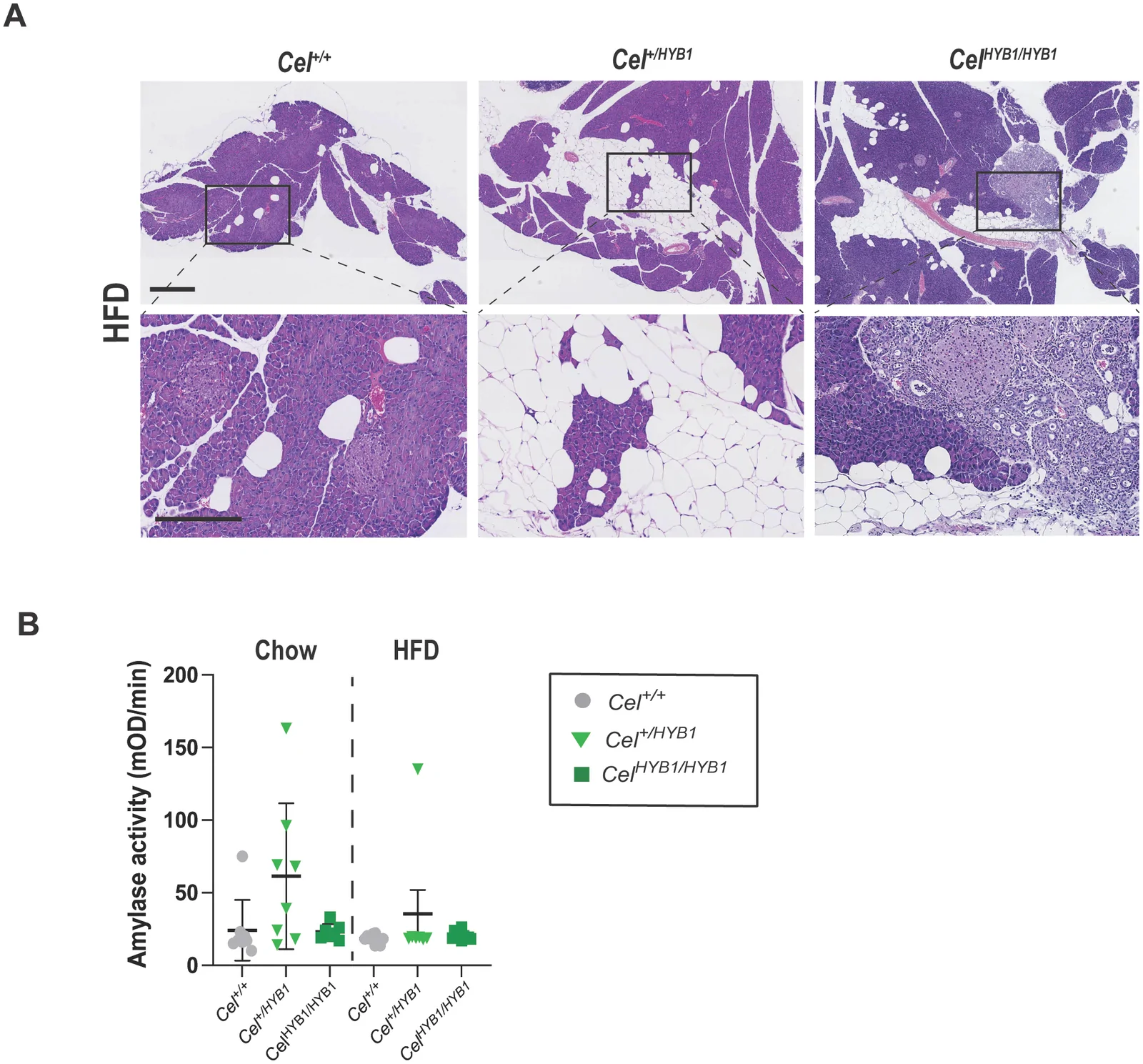
Pancreas histology and amylase activity in *Cel-HYB1* male mice on HFD. **(A)** Pancreas histology of *Cel^+/+^*, *Cel^+/HYB1^* and *Cel^HYB1/HYB1^* fed HFD. The images show examples of histological abnormalities including inflammation, fatty replacement and acinar atrophy observed in the mouse groups. An affected animal did not necessarily present with all pathological features, but could for example exhibit only fatty replacement, like in the left and middle images. Scale bars are 500 µm (for images with low magnification) and 250 µm (high magnification). **(B)** Serum amylase activity was measured in *Cel^+/+^*, *Cel^+/HYB1^* and *Cel^HYB1/HYB1^* mice on chow diet or HFD. Individual values are shown with mean (horizontal bar) ± SD.

#### 3.2.3 Pancreatic CEL protein expression

To evaluate CEL protein distribution, pancreatic tissue sections from *Cel^+/+^* and *Cel^HYB1/HYB1^* mice were analyzed by immunohistochemistry. Male and female mice of both diet groups generally showed the same pattern: CEL was present as brown puncta consistent with zymogen granules, and the protein was equally distributed throughout the tissue. However, *Cel^HYB1/HYB1^* mice on HFD exhibited irregular and more intensely stained CEL-positive structures in atrophic areas (**Figure 6**), consistent with protein aggregation. Outside the atrophic regions, the staining pattern was comparable between genotypes.

**Figure 6:**
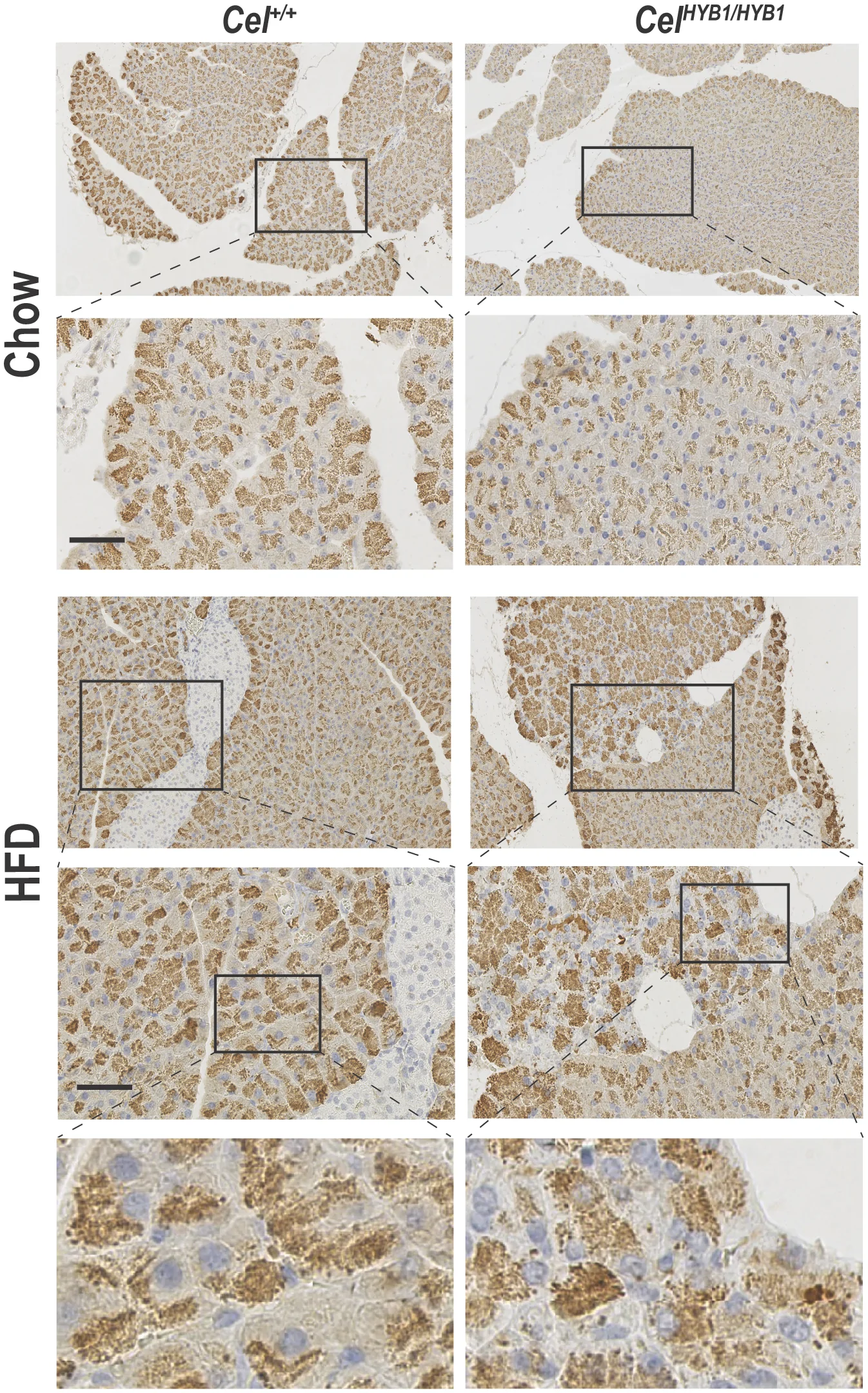
CEL protein expression in the pancreas of *Cel-HYB1* male mice on HFD. Pancreatic tissue sections from *Cel^+/+^* and *Cel^HYB1/HYB1^* mice on chow diet (upper panel) or HFD (lower panel) underwent chromogenic staining with an anti-CEL antibody. Representative images are shown. Scale bars are 60 µm.

#### 3.2.5 Endocrine function

Random blood glucose measurements in mice fed HFD or control diet did not indicate impaired glucose homeostasis at any time between 6 and 16 weeks of age (data not shown). However, IPGTT performed at 15 weeks revealed clearly elevated glucose levels in all groups of HFD-fed mice compared to their chow-fed control groups (**Figures 7A, B**). This was expected since obesity can lead to insulin resistance (Kahn and Flier, 2000). Interestingly, there was a general tendency for both diets that *Cel^+/HYB1^* and *Cel^HYB1/HYB1^* mice were slightly more glucose-tolerant than wildtype controls, although only in a couple of comparisons did the difference reach statistical significance (**Figure 7B**).

**Figure 7:**
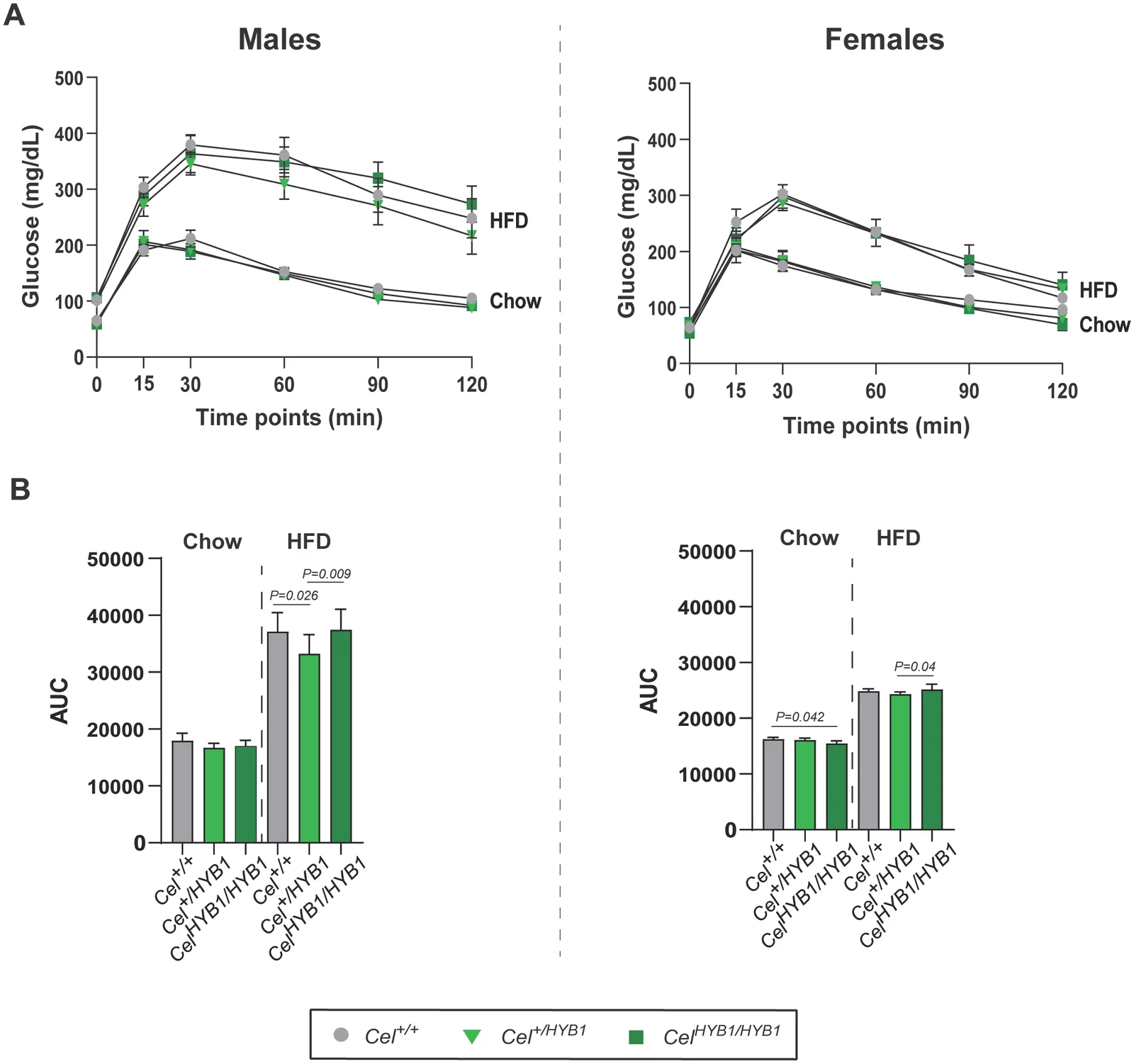
Glucose homeostasis in *Cel-HYB1* male and female mice on HFD. **(A)** Intraperitoneal glucose tolerance test of *Cel^+/+^*, *Cel^+/HYB1^* and *Cel^HYB1/HYB1^* mice on chow diet or HFD. **(B)** Comparison of area under the curve (AUC) for the mice in **(A)**. Results are shown as mean ± SEM. *P*-values are listed for statistically significant differences between groups.

## 4 Discussion

Chronic pancreatitis is a multifactorial disease in which genetic risk most often operates in combination with lifestyle and anatomical factors (Kleeff et al., 2017; Gardner et al., 2020). The *CEL-HYB1* allele is an established genetic risk factor for CP (Fjeld et al., 2015; Berke et al., 2024), and analysis of two Norwegian families suggested that *CEL-HYB1* can act in concert with cigarette smoking and pancreas divisum to increase disease risk (Tjora et al., 2021). The pathogenicity of *CEL-HYB1* has been confirmed by two independent mouse models that spontaneously develop disease, one made by us (Fjeld et al., 2022) and the other by a Chinese group (Mao et al., 2022). In the current study, we examined how genetic context and metabolic stress may influence the pancreatic phenotype in *Cel-HYB1* mice.

We first asked whether the normal CEL protein with 16 VNTR repeats could influence the pathogenicity of *Cel-HYB1*. Previous cell culture experiments suggested that the toxic effect of human CEL-HYB1 may be reduced by co-expression with normal CEL protein (Dalva et al., 2020). In heterozygous *Cel-HYB1* mice, however, the normal, endogenous mouse *Cel* gene contains only three VNTR repeats (Fjeld et al., 2022). We therefore developed compound heterozygous *Cel^16R/HYB1^* mice expressing two humanized knock-in alleles, one with the pathogenic *CEL-HYB1* VNTR and one with the normal 16R VNTR, thereby mimicking the genetic constellation of *CEL-HYB1* carriers.

Several differences were observed between *Cel^+/HYB1^* and *Cel^16R/HYB1^* mice; however, they did not form a consistent genotype-dependent pattern. Male *Cel^16R/HYB1^* mice had increased white adipose tissue mass and tended to have higher body weights than their *Cel^+/HYB1^* littermates, whereas the opposite trend in body weight was observed in females. *Cel^16R/HYB1^* mice also displayed improved glucose tolerance compared with *Cel^+/HYB1^* mice. Histological evaluation, however, did not indicate a more severe pancreatic phenotype in *Cel^16R/HYB1^* mice (**Table 1**). Rather, atrophy was more prominent in male *Cel^+/HYB1^* mice, while fatty replacement was observed in a greater proportion of female *Cel^+/HYB1^* mice than in their *Cel^16R/HYB1^* counterparts. Taken together, the observed differences were modest and varied according to sex and phenotypic feature, providing no evidence of a consistent modifying effect of the 16R allele on the pancreatic phenotype. Still, as CEL mRNA and protein levels were not assessed, we cannot exclude that altered CEL expression according to type of VNTR may have impacted our observations.

In parallel, *Cel-HYB1* mice were challenged with HFD feeding from 6 to 16 weeks of age. The increased frequency of mild histopathological changes in *Cel-HYB1* mice (15/24) on HFD compared to control mice (3/12) supports the notion that metabolic stress and obesity may aggravate the effects of this genetic risk factor (**Table 2**). Similar alterations were observed in a subset of control mice, indicating that HFD alone can induce pancreatic tissue changes, albeit to a lesser extent than in the genetically modified mice. Surprisingly, HFD did not affect amylase activity in *Cel-HYB1* mice despite the presence of histopathological changes (**Figure 5**). This likely reflects the narrow temporal window of serum amylase elevation during acute pancreatic injury (Ismail and Bhayana, 2017), limiting its sensitivity in detecting chronic or mild disease.

As expected, HFD feeding resulted in impaired glucose tolerance compared to chow-fed animals. Genotype-associated differences in glucose homeostasis were generally small or absent (**Figure 7**). Notably, however, a general trend toward slightly improved glucose tolerance was observed in *Cel-HYB1* mice, indicating that the presence of the pathogenic allele does not impair—and may even slightly improve—glucose handling in mice. This observation is consistent with previous studies showing that endocrine dysfunction is rarely observed in genetic mouse models of chronic pancreatitis (Geisz and Sahin-Toth, 2018; Zhu et al., 2023). One instructive example is a recently developed mouse model for the human disease *CEL-MODY* (also known as MODY8), a hereditary syndrome of diabetes and chronic pancreatitis (Gravdal et al., 2025). Although the pathogenic *CEL-MODY* variant has a considerably stronger effect than *CEL-HYB1* (Johansson et al., 2018), the *Cel*-MODY mouse model exhibited somewhat improved glucose tolerance compared to controls (Gravdal et al., 2025). The lack of overt diabetes in mouse CP models may be rooted in the many physiological and anatomical differences between the murine and human pancreas (Dolensek et al., 2015).

Our initial study of *Cel-HYB1* mice included an analysis of adipose tissue and revealed sex-specific differences, with male animals displaying larger fat depots than females (Fjeld et al., 2022). Such sex-dependent differences were less pronounced in the *Cel^16R/HYB1^* model. Nevertheless, male, but not female *Cel^16R/HYB1^* mice showed a significant increase in gonadal white adipose tissue compared with *Cel^+/HYB1^* mice (**Figure 1**). Histopathological changes in the pancreas were more frequently observed in male than in female *Cel-HYB1* mice in the HFD study (**Table 2**). In contrast, at 12 months of age, female *Cel^+/HYB1^* and *Cel^16R/HYB1^* mice generally exhibited more pronounced pancreatic histopathological changes than their male counterparts (**Table 1**). Taken together, our data indicate that sex may influence several aspects of the *Cel-HYB1* mouse phenotype, although the direction and magnitude of the differences appear to depend on age, genetic context, and experimental conditions. Sex differences in pancreatitis have also been reported in humans; however, the available studies are limited, and the underlying mechanisms remain poorly understood (Drake et al., 2021; Wang et al., 2021).

Another observation related to sex difference is linked to endocrine function as *Cel-HYB1* male mice showed impaired glucose tolerance at 12 months (Fjeld et al., 2022). We have later repeated the experiment in 12-month-old *Cel-HYB1* females, and they presented with improved glucose tolerance (**Supplementary Figure 3**), like the female *Cel^16R/HYB1^* and *Cel-HYB1* mice on chow diet in this study (**Figures 3**, **7**). Comparable sex differences have been reported in other mouse models of diabetes; for instance, in streptozotocin-induced diabetes, females show lower susceptibility to hyperglycemia than males (Deeds et al., 2011). In fact, it has been suggested that estrogen may exert protective effects against the development of diabetes (Le May et al., 2006; De Paoli and Werstuck, 2020).

A central mechanistic question is whether HFD acts specifically on CEL expression, thereby increasing the production of the misfolded enzyme in pancreatic acinar cells. Lipids are potent stimulators of pancreatic enzyme secretion. In humans, increased pancreatic enzyme output induced by a fat-rich diet primarily involves trypsin and amylase, whereas lipase secretion is modestly affected (Boivin et al., 1990). In contrast, several older studies in rats demonstrated that pancreatic lipase levels increased significantly when the dietary fat content was raised (Gidez, 1973). Moreover, Birk et al (Birk et al., 2014). examined the mRNA levels of various digestive enzyme genes in the mouse pancreas in response to chronic HFD feeding. Transcripts encoding pancreatic lipases (PNLIP, PLRP2, PLA2) were initially elevated but declined to basal control levels by the end of the study. Notably, the transcript level of CEL was not significantly affected (Birk et al., 2014).

We therefore believe that the most plausible scenario is that HFD does not exert a specific effect on CEL expression. Rather, this diet may act as a general metabolic stressor of the pancreas, although the response regarding types of digestive enzymes may vary among species. HFD may also promote pathogenic processes in other mouse models of pancreatitis. For example, mice expressing the human PRSS1 R122H mutation developed more severe pancreatitis than mice expressing wildtype human PRSS1 when the animals were fed HFD (Huang et al., 2020). Furthermore, a study focusing on pancreatic fat in normal mice demonstrated that HFD accelerated not only intrapancreatic fat accumulation but also cerulein-induced fibrosis and inflammation, i.e. hallmarks of pancreatitis (Yin et al., 2026).

In summary, our study provides further insight into how the *CEL-HYB1* risk allele contributes to pancreatic disease. We show that the normal humanized *Cel-16R* allele does not substantially modify the pancreatic phenotype associated with *Cel-HYB1*, at least not under basal conditions, whereas metabolic stress seems to aggravate the exocrine phenotype. In addition, our findings point to potential sex-dependent differences.

More broadly, our results support the view that CP is a multifactorial disease in which genetic risk factors such as *CEL-HYB1* act in concert with environmental exposures and biological modifiers. A comprehensive understanding of pancreatitis pathogenesis therefore requires an integrated approach that considers gene–gene and gene–environment interactions, as well as sex-specific effects. Future studies addressing these interactions at both the molecular and physiological level will be important to elucidate the mechanisms underlying *CEL-HYB1*-associated CP.

## Data Availability

The raw data supporting the conclusions of this article will be made available by the authors, without undue reservation.

## Supplementary material

Supplementary Figure 1 Genetic verification of mouse strains. Genotyping of control (*Cel^+/+^*), heterozygous (*Cel^+/16R^*), heterozygous (*Cel^+/HYB1^*), compound heterozygous (*Cel^16R/HYB1^*) and homozygous (*Cel^HYB1/HYB1^*) mice were performed by PCR as described in Section 2.3. The PCR products were visualized by agarose gel electrophoresis as shown in the image.

Supplementary Figure 2 Absolute pancreas weight (g) in *Cel^+/HYB1^* and *Cel^16R/HYB1^* male and female mice at 6 and 12 months of age. Individual values with mean (horizontal bar) ± SD are shown.

Supplementary Figure 3 Glucose homeostasis in 12-months-old *Cel-HYB1* female mice. **(A)** Intraperitoneal glucose tolerance test of female *Cel^+/+^*, *Cel^+/HYB1^* and *Cel^HYB1/HYB1^* mice. **(B)** Comparison of area under the curve (AUC) for the mice in **(A)**. Results are shown as mean ± SEM. *P*-values are listed for statistically significant differences between groups.
